# Properties of Styrene–Maleic Anhydride Copolymer Compatibilized Polyamide 66/Poly (Phenylene Ether) Blends: Effect of Maleic Anhydride Concentration and Copolymer Content

**DOI:** 10.3390/ma13051237

**Published:** 2020-03-09

**Authors:** Alper Aksit, Teresa Menzel, Merve Aksit, Volker Altstädt

**Affiliations:** 1 Department of Polymer Engineering, University of Bayreuth, Universitätsstraße 30, 95447 Bayreuth, Germany; alper.aksit@uni-bayreuth.de (A.A.); teresa.menzel@uni-bayreuth.de (T.M.); merve.aksit@uni-bayreuth.de (M.A.); 2 Bavarian Polymer Institute and Bayreuth Institute of Macromolecular Research, University of Bayreuth, Universitätsstraße 30, 95447 Bayreuth, Germany

**Keywords:** blend, compatibilization, SMA, PA66, PPE, melt blending, morphology, tensile

## Abstract

Polyamide 66 (PA66)/poly (2,6-dimethyl-1,4-phenylene ether) (PPE) blends with a ratio of 50/50 (w/w) were produced by a twin-screw compounder. The immiscible blends were compatibilized using two different styrene–maleic anhydride copolymers (SMA) with a low (SMA_low_) and a high (SMA_high_) maleic anhydride (MA) concentration of 8 and 25 wt%, respectively. Furthermore, the SMA content was varied from 0 to 10 wt%. The influence of MA concentration and SMA content on the morphological and thermomechanical properties of PA66/PPE blends was investigated. Herein, we established correlations between the interfacial activity of the SMA with blend morphology and corresponding tensile properties. A droplet-sea to co-continuous morphology transition was shown by scanning electron microscopy to occur between 1.25 and 5 wt% in the case of SMA_high_. For SMA_low_, the transition started from 7.5 wt% and was still ongoing at 10 wt%. It was found that SMA_low_ with 10 wt% content enhanced the tensile strength (10%) and elongation at break (70%) of PA66/PPE blends. This improvement can be explained by the strong interfacial interaction of SMA_low_ within the blend system, which features the formation of nanoemulsion morphology, as shown by transmission electron microscopy. Very small interdomain distances hinder matrix deformations, which forces debonding and cohesive failure of the PPE phase as a “weaker” main deformation mechanism. Due to a lack of interfacial activity, the mechanical properties of the blends with SMA_high_ were not improved.

## 1. Introduction

Polymer blending technology has evolved to be a convenient way for the production of new polymers by using conventional processing methods [1,2,3,4]. The major benefit of blending is combining the properties of each component. However, most of the polymer/polymer combinations are immiscible and thus tend to macrophase separate when mixed [5,6,7,8,9]. Phase separation leads to the formation of weak interfaces and cause high stress concentrations locally when under load. In order to control the phase separation, various compatibilization methods are applied, such as the addition of low-molecular-weight organic molecules [10,11,12,13], inorganic nanoparticles [14,15,16,17], Janus-type hybrid materials [18,19,20,21], and copolymers with functional moieties [22,23,24,25,26].

Due to its good processability, oil, and heat resistance, polyamide (PA) is widely used in engineering applications, such as automotive and electronics. However, several drawbacks, such as high moisture absorption and poor dimensional stability, limit its use. Due to its dimensional stability, water, and temperature resistance, poly (2,6-dimethyl-1,4-phenylene ether) (PPE) is a promising blend partner to overcome the mentioned drawbacks of PA. As both polymers are immiscible when blended, compatibilization is needed to improve the interfacial interaction and thus the mechanical properties [27]. Copolymer compatibilizers are frequently used for enhancing the properties of PA6/PPE blends. The compatibilization mechanisms of copolymers are based on a physicochemical approach. On the one hand, they form a covalent bond with PA6 by the chemical reaction between the copolymer moieties and either amine or carboxyl moieties of the PA6. On the other hand, a physical affinity, particularly chain entanglements of the PPE and the compatibilizer backbone, are observed [28,29,30,31]. Amongst copolymer-type compatibilizers, styrene–maleic anhydride copolymers (SMA) is the most commonly used for PA6/PPE blends [32,33,34,35]. Low-priced monomers and facile synthesis via radical polymerization enable a broad commercial availability of SMA, having different MA concentrations. With changing MA concentration, the solubility of SMA in PPE is altered. A miscibility limit of SMA in PPE is found at an MA concentration of 8 wt% [36,37]. The effect of MA concentration in SMA when blended with PA6/PPE was also studied. An MA concentration of 8 wt% in SMA is found to be more beneficial than SMA with 2 wt% MA in terms of reduction of the PPE domain sizes [38]. For many years, it was expected that SMA with MA concentrations higher than 8 wt% would not reveal interfacial activity and thus not improve the mechanical properties of PA6/PPE blends. A study was published by Wang et al. [39] where SMA with 21.8 wt% MA was used as a compatibilizer. Even though it is known that this SMA type is insoluble in PPE, the authors claim that interfacial interactions exist between the SMA and the PPE phase, resulting in a decrease in the mean size of the PPE domains. Interestingly, the researchers compared their tensile results with the study of Chiang and Chang [35] and claimed that SMA with 21.8 wt% MA is more efficient than the SMA (8 wt% MA) used by Chiang and Chang in terms of tensile strength. For a constant PPE/PA6 blend ratio of 30/70 (w/w), it is stated that the tensile strength increases up to 70.7 MPa (starting from 47.6 MPa at 0 wt% SMA) with the addition of 10 wt% SMA. Subsequently, it was concluded that the authors of [35] only increased the tensile strength from 29 to 44 MPa with the equivalent amount of 10 wt% SMA (8 wt% MA concentration). In absolute numbers, Wang et al. achieved higher values for tensile strength; nevertheless, relative numbers reveal an increase of 48.5% for Wang et al. and 51.7% for Chiang and Chang. With knowing this, the role of the MA concentration (especially at concentrations higher than 8 wt%) on the mechanical performance of PA6/PPE/SMA has not been studied systematically.

Recently, the research with PA66-based PPE blends as an alternative to PA6 has been gaining more attention as PA66 features superior mechanical performance, especially at elevated temperatures. Nonetheless, new challenges come out with the use of PA66 due to its higher reactivity and sensitivity to hydrolysis compared to PA6.

Most of the studies in PA66/PPE blends mainly focus on low-molecular weight organic components for compatibilization [40,41,42,43,44]. For the first time, Kim et al. [45] used SMA to investigate the compatibilization efficiency with a poly (styrene-b-ethylene/butylene-b-styrene)-g-MA (SEBS-MA) copolymer for PA66/PPE/high-impact polystyrene (HIPS) (75/12.5/12.5 w/w/w) ternary blend. They varied the ratio between both compatibilizers and analyzed the compatibilizer ratio on tensile and impact properties. Using only 20 wt% SMA (on given ternary blend composition) leads to an increase in tensile strength of 50%, with no significant changes for elongation at break and impact strength. A better compatibilization was achieved by smaller domain sizes of the PPE/HIPS.

To the best of our knowledge, a fundamental investigation on SMA-compatibilized PA66/PPE blends is completely missing in the literature. Therefore, in this study, we aim to gain a deep understanding of the influence of SMA copolymers on the properties of a PA66/PPE blend (50/50 w/w). Structure–property relationships between blend morphology, controlled by different MA concentrations and SMA contents and mechanical properties, are established.

## 2. Materials and Methods

### 2.1. Materials

A commercially available PA66 compounding grade, PPE powder, and SMA with various MA concentrations were used as provided. Most of the relevant material properties are shown in Table 1. The weight-averaged molecular weight (M_w_) and polydispersity measurements of PA66 and PPE were performed via gel permeation chromatography (GPC). An instrument having four PSS-SDV gel columns (particle size = 5 µm) with a porosity range from 102 to 105 Å (PSS, Mainz, Germany) using a nonselective refractive index detector (Shodex, Techlab, Japan). Hexafluoroisopropanol (HFIP) and chloroform (CHCl_3_) were used as eluents for PA66 and PPE, respectively. The eluent flow rate was set at 1.0 mL/min. The calibrations for PA66 and PPE were done with poly(methyl methacrylate) and narrowly-distributed polystyrene (PS) for PA66 and PPE, respectively.

### 2.2 Processing of Blends

Prior to processing, PA66 was dried overnight by using a dry-air granulate dryer (TLE 100, Gerco Technik GmbH, Enningerloh, Germany) at 80 °C. All materials were compounded at 270 °C and 300 rpm in a co-rotating twin-screw compounder (ZSK 26 MCC, Coperion GmbH, Stuttgart, Germany). Pellets were obtained by strand pelletizing after passing a water bath. For binary blends of PA66 and PPE, a single-step compounding was applied. The ternary blends of PA66, PPE, and SMA were melt-blended via two-step processing, where PA66/SMA blends were reactively compounded in the first stage. After overnight drying at 80 °C, the pellets were melt-blended with PPE.

The PA66/PPE blend ratio was set constant at 1:1 (w/w). Based on this, the SMA contents were varied from 1.25, 5, 7.5 to 10 wt%.

Specimens of overnight dried blends were prepared by injection molding (Arburg Allrounder 470H 1000-170, Arburg GmbH, Loßburg, Germany) with different geometries for further characterization. A nozzle and mold temperature of 290 °C and 100 °C and a cooling time of 20 s were applied.

### 2.3. Dynamic–Mechanical Analysis (DMA)

For the investigation of thermomechanical properties, a Gabo Eplexor 500N (NETZSCH-Gerätebau GmbH; Selb, Germany) DMA was used in tensile mode. Oscillatory stress (2.5 MPa) was applied at a frequency of 1 Hz while heating from 25 to 255 °C at a heating rate of 2 K/min. For the evaluation of the glass transition, tan δ values were plotted against temperature, wherein the peak values were considered. Each measurement was repeated three times to minimize the experimental errors.

### 2.4. Morphological Characterization

Morphological analysis was done via a field-emission scanning electron microscopy (FESEM) Zeiss LEO 1530 (Zeiss NTS GmbH, Oberkochen, Germany) at an acceleration voltage of 3 kV. The surfaces of cyrofractured tensile bars were etched with CHCl_3_ to selectively dissolve the PPE phase.

Further analysis was carried out via transmission electron microscopy (TEM) at an acceleration voltage of 200 kV using a Zeiss EM922 OMEGA (Zeiss NTS GmbH, Oberkochen, Germany). Ultrathin sections (approximately 60 nm) were prepared from injection molded tensile bars using an ultra-microtome (Leica EM UC7, Leica Microsystems GmbH, Wetzlar, Germany) equipped with a diamond knife. The ultrathin sections were stained with ruthenium tetroxide for 15 min in order to enhance the contrast between the two major phases. The number-averaged PPE domain sizes were calculated from 100 droplets considering the largest diameter for each domain, as the shapes were rather ellipsoid. Here, we assume that the cuts have gone through the middle of each domain.

Fractographs after tensile testing were taken at an acceleration voltage of 3 kV. Representative tensile bars, with values closest to the average were sputtered with platinum (1.3–2 nm thickness) prior to the measurements.

### 2.5. Mechanical Characterization

Tensile testing was performed using a universal testing machine (Zwick Z020, ZwickRoell GmbH & Co. KG, Ulm, Germany) equipped with an extensometer. The measurements were conducted according to ISO 527-2 using 1A type specimen [47]. Since PA66 is hygroscopic, all samples were dried overnight at 80 °C under vacuum and subsequently vacuum-sealed to guarantee the absence of humidity. The samples were taken out of the sealed bags prior to the measurements.

## 3. Results and Discussion

### 3.1. Miscibility of SMA in PPE

**Figure 1:**
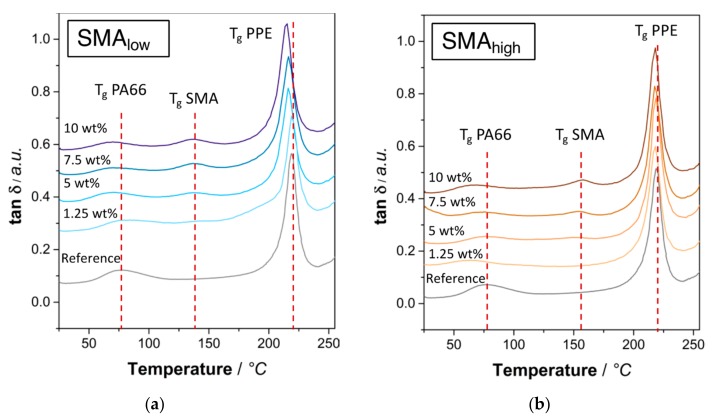
Tan δ plots of SMA compatibilized PA66/PPE (1:1 w/w) ternary blends with (**a**) SMA_low_, (**b**) SMA_high_ at various contents.

To evaluate the miscibility of two polymers various methods, such as differential scanning calorimetry (DSC), DMA, or SEM/TEM can be applied. In DMA measurements, tan δ plots depict an easy method for the determination of thermal transitions. For non-miscible binary blends, two distinct peaks are visible, indicating independent transitions of each polymer. For miscible polymer blends, the two peaks coincide to obtain a single signal in between the two individual signals depending on the blend ratio [48]. For interacting polymers, either both peaks approach each other or one of the peaks reveals a shift. In Figure 1a,b the tan δ versus temperature plots are given for SMA_low_ and SMA_high_, respectively.

Starting from 5 wt% SMA_low_ content (Figure 1a), a shift of the PPE glass transition temperature (T_g_) at 219 °C is seen. At 10 wt%, a maximum shift of −4 °C is observed. This indicates an interaction between SMA_low_ and PPE; however, it cannot absolutely be stated that SMA_low_ is (partially) miscible in the PPE phase. The occurrence of the SMA peak at 130 °C agrees with the literature [49]. For SMA_high_ (Figure 1b), no significant shift of the PPE peak is visible, indicating that neither an interaction nor a miscibility with PPE exists. The SMA_high_ peak at 155 °C appears higher than the peak of SMA_low_, which is given by its higher concentration of MA.

Furthermore, it is observed that the T_g_ of PA66 is shifted to lower values when either of the SMA is added. Since the reaction of anhydrides and amines eliminates water, PA66 is likely to hydrolyze. As a result, local chain scission of the PA66 lowers the M_w_ and thus the T_g_ [50]. The T_g_ signals of the reference and 10 wt% SMA_low_ and SMA_high_ are given in Table 2.

According to Table 2, higher MA concentrations result in a more pronounced peak shift in the PA66 signal. The MA concentration is proportional to the amount of water released by the anhydride–amide reaction, thus leading to a higher degree of chain scission.

**Figure 2:**
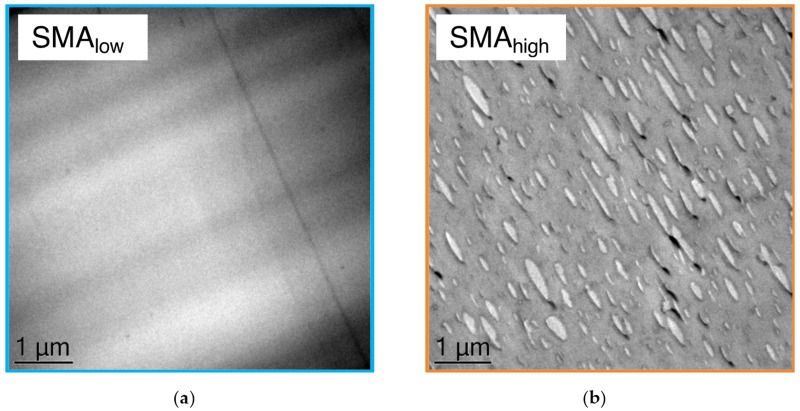
TEM micrographs of (**a**) PPE/SMA_low_ and (**b**) PPE/SMA_high_ binary blends with ratios of 82/18 (w/w).

To approve the DMA results and further clarify the miscibility of both SMA in the PPE, TEM micrographs were taken from PPE/SMA binary blends with a PPE/SMA ratio of 82/18 (w/w) as shown in Figure 2a,b.

For SMA_low_ (Figure 2a), no phase contrast, i.e., no phase separation is visible, which can be explained by a complete miscibility of SMA_low_ with PPE.

Figure 2b indicates a distinct phase separation of the SMA_high_ (minor phase, shown in light gray) and PPE (major phase, shown in dark gray), resulting in a droplet-sea morphology. This strong phase separation is induced by the mismatch of the two components due to the high polarity of SMA_high_, leading to elongated large SMA_high_ domains. These domains (diameter range from 400 to 1200 nm) result in a non-transparent binary blend.

### 3.2. Blend Morphology

**Figure 3:**
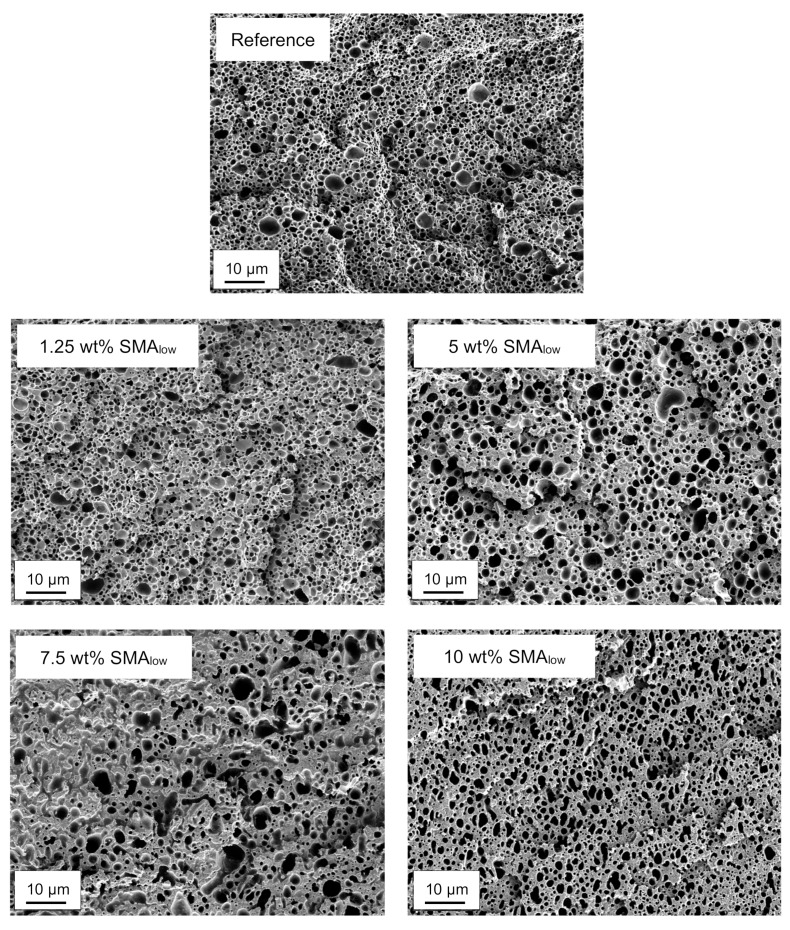
SEM micrographs of selectively etched PA66/PPE/SMA_low_ ternary blends.

Figure 3 shows the SEM micrographs of selectively etched PA66/PPE blends with 1.25, 5, 7.5, and 10 wt% SMA_low_ content together with the reference.

According to Figure 3, up to 5 wt% SMA_low_ blends show droplet-sea morphology. For 1.25 wt%, a qualitative reduction of the PPE domain size distribution is visible; however, it is coarsening beyond the distribution of the neat system for 5 wt% SMA_low_. Interestingly, a further increase in SMA_low_ content to 7.5 wt% and 10 wt% cannot reduce the domain size of the PPE phase. The coarsening of the PPE domains has a maximum at 7.5 wt% SMA_low_, as for 10 wt%, a finer morphology is seen.

The irregular domains induced by 7.5 and 10 wt% SMA_low_ indicate an incomplete transition from droplet-sea to co-continuous morphology.

**Figure 4:**
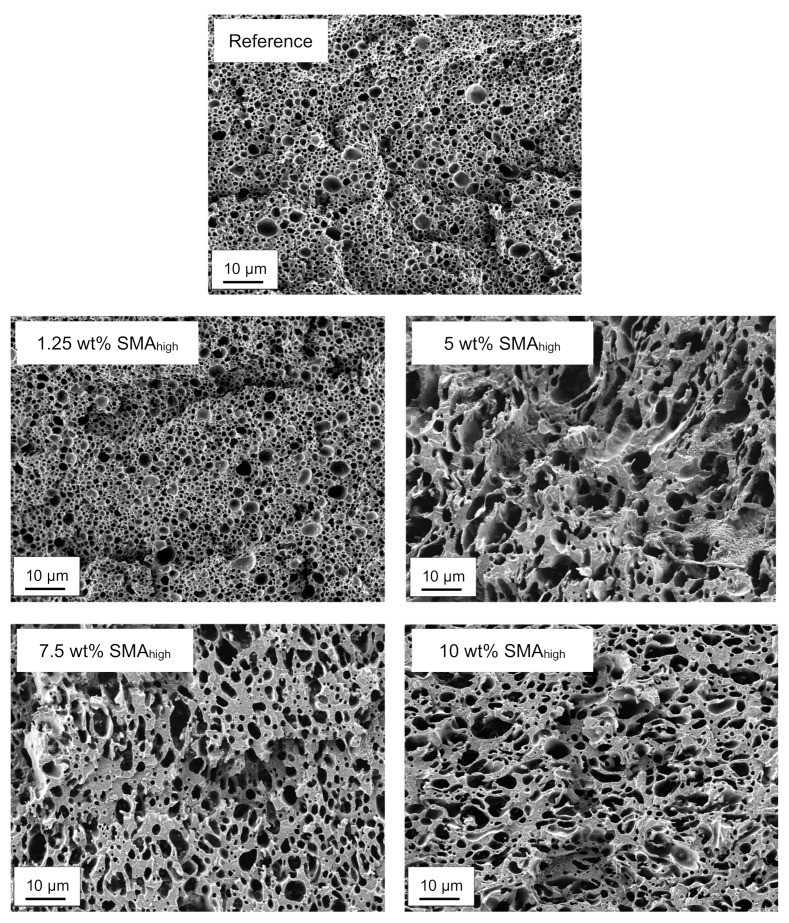
SEM micrographs of selectively etched PA66/PPE/SMA_high_ ternary blends.

Figure 4 exhibits the SEM micrographs of PA66/PPE blends with 1.25, 5, 7.5 and 10 wt% SMA_high_ content together with the reference.

In contrast to SMA_low_, an earlier droplet-sea to co-continuous transition for SMA_high_ between 1.25 and 5 wt% starts (Figure 4). As observed for SMA_low_, further SMA_high_ addition cannot decrease the domain size of the PPE phase. For 7.5 wt% SMA_high_, the transition proceeds and finishes at the maximum concentration of 10 wt% SMA_high_. As seen for SMA_low_, a coarsening of the PPE phases is obvious for SMA_high_ blends, with a maximum at 5 wt%. A further increase of SMA_high_ content results in a decrease of the PPE domain sizes and a more homogeneous structure.

The earlier transition of SMA_high_ is explained by the shift of the viscosity ratio of PA66/PPE to lower values. The viscosity ratio λ stated by Utracki is given in Equation (1), where η_d_ is the viscosity of the dispersed phase (PPE) and η_m_ is the viscosity of the matrix phase (PA66) [2].

λ = η_d_/η_m_ (1)

For a constant blend ratio of 50/50 PA66/PPE, a large λ value is calculated from Equation (1) due to the high intrinsic viscosity of PPE and low viscosity of PA66, which justifies the visible droplet-sea morphology with PPE domains dispersed in a PA66 matrix. With the introduction of SMA_high_ into PA66, the viscosity of the binary blend (PA66/SMA_high_) increases. With the addition of a sufficient amount of SMA, λ approaches a value close to 1, where equally viscous polymers tend to form co-continuous structures during melt blending.

**Figure 5:**
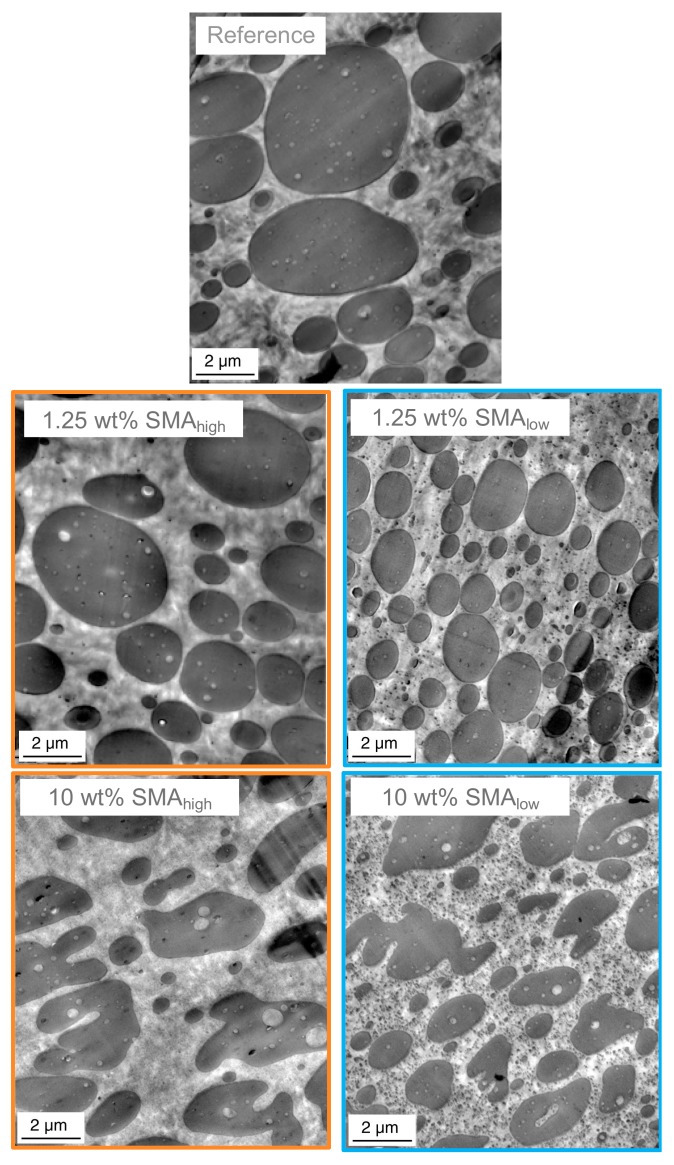
TEM micrographs of SMA compatibilized PA66/PPE ternary blends, with SMA_high_ (left column) and SMA_low_ (right column).

As shown in Figure 3 and Figure 4 both SMA_low_ and SMA_high_ lead to droplet-sea morphology for 1.25 wt%. A co-continuous morphology is only observed for 10 wt% SMA_high_ with a transition between 1.25 and 7.5 wt% and co-continuous morphology for 10 wt% SMA content. To validate the morphological interpretations via SEM, TEM micrographs of the reference and blends with 1.25 and 10 wt% SMA (low and high) are shown in Figure 5.

As expected, the blends with 1.25 wt% SMA (low and high) possess a droplet-sea morphology, while the blends with 10 wt% SMA contain irregularly shaped PPE domains. From Figure 4, we know that the 10 wt% SMA_high_ compatibilized blend forms a bi-continuous phase separation.

Interestingly, with 1.25 wt% SMA (high and low), changes within the PA66 matrix are noticeable and even more pronounced at 10 wt%.

**Figure 6:**
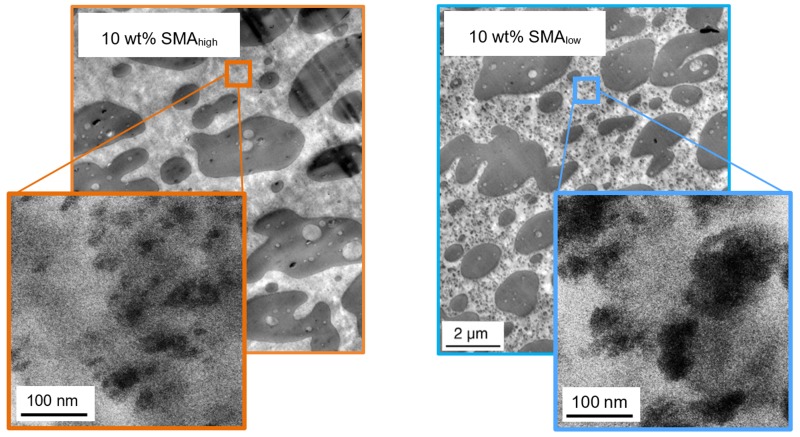
TEM micrographs of PA66/PPE blends with 10 wt% SMA_high_ (**left**) and SMA_low_ (**right**).

The apparent changes within the PA66 phases are highlighted by TEM micrographs at higher magnifications for 10 wt% SMA (low and high) given in Figure 6.

Herein, sub-micron sized black spots are distributed all over the matrix having a diameter of 100 nm and smaller in size for both SMA. The size distribution of the small inclusions together with the PPE domain size distribution of the reference without SMA is given in Table 3.

Table 3 shows that the nano-sized inclusions are approximately 10 times smaller than the large PPE domains. Interestingly, the inclusions with SMA_low_ are four times larger compared to the inclusions of SMA_high_. As already discussed in Section 3.1, SMA_high_ is not miscible with PPE due to its high polarity. This allows us to conclude that the matrix inclusions consist of SMA_high_-g-PA66 copolymer and possibly unreacted SMA_high_ micelles. In contrast, the nano-inclusions of the ternary blend with SMA_low_ seem to be swollen. We propose that these micelles have a core-shell like structure with either a SMA_low_-g-PA66 or unreacted SMA_low_ shell and a PPE core. The balanced polarity of the SMA_low_ enables a strong interfacial interaction within the PA66/PPE blend. With sufficient SMA_low_ content, all interfaces between PA66 and PPE are saturated, and thus, the interfacial tension of the blend system is minimized. Consequently, the surface roughening of PPE followed by pinch-offs occurs, enabling SMA_low_ to diffuse to the newly generated interfaces. The phenomenon of micelle formation and emulsification was already described for other blend systems by several work groups [51,52,53].

### 3.3. Tensile Properties

**Figure 7:**
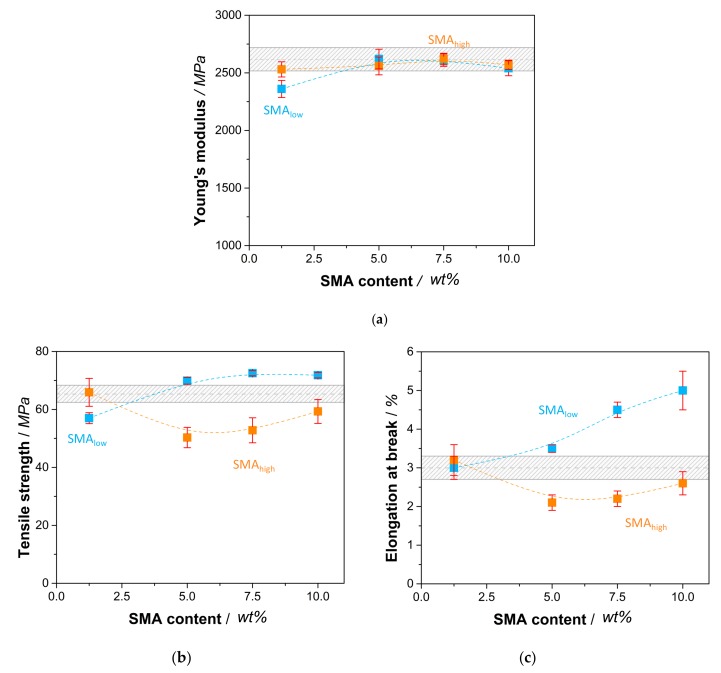
Tensile properties of SMA compatibilized PA66/PPE blends with Young’s modulus (**a**), tensile strength (**b**), and elongation at break (**c**). All properties are plotted against the SMA content with SMA_low_ (blue rectangular) and SMA_high_ (orange rectangular). Gray bars represent the values of the PA66/PPE binary blend without SMA.

The tensile properties of SMA compatibilized PA66/PPE blends are shown in Figure 7a–c wherein the Young’s modulus (E) is displayed together with the tensile strength (σ_m_) and elongation at break (ε_b_), respectively.

In comparison to the reference blend, Figure 7a shows a reduction in Young’s modulus with 1.25 wt% SMA_low_ (2360 MPa), whereas a recovery is seen with a maximum at 5 wt% (2620 MPa). The further incorporation of SMA_low_ causes lower modulus values of 2540 MPa (10 wt%), which still is in the range of the reference. For SMA_high_, the modulus is found to be independent of the SMA content, showing no significant change.

For σ_m_ (Figure 7b) and ε_b_ (Figure 7c), the influence of SMA content is more pronounced. For SMA_low_, 1.25 wt% leads to deteriorated properties due to the disordered interfaces between PA66 and PPE, increasing local stress concentrations. However, we would expect better mechanical performance as the PPE domain sizes decrease (Figure 3), indicating a reduction of interfacial tension and successful compatibilization [25]. The further addition of SMA_low_ results in an increase of σ_m_ up to 11 % for 7.5 wt%, facing a plateau with no further increase at 10 wt%. It seems that the occurring morphology transition at 5–10 wt% (Figure 3) competes with the compatibilization effect of SMA_low_, resulting in only moderate improvements.

With 1.25 wt% SMA_low_, the elongational properties of the blend do not differ from the reference. Starting from 5 wt%, a constant increase in elongation at break is observed, reaching its maximum at 10 wt% with a total increase of 70% compared to the reference. From Section 3.2, it is known that the morphology transition from droplet-sea to co-continuous structures happens at 5 and 10 wt% SMA_low_. As for tensile strength results, a change in morphology seems to overpower the compatibilization effect, leading to detrimental tensile properties compared to a clear droplet-sea type of phase separation. This phenomenon is also valid for the results of SMA_high_ compatibilized blends. For 1.25 wt% SMA_high_, values of σ_m_ and ε_b_ are within the range of the reference. With the transition to co-continuous structures, a decrease in both σ_m_ and ε_b_ is seen at 5 wt% SMA_high_. Interestingly, the partial recovery of both values is evident for higher amounts of SMA_high_. These results agree with the findings of [34,35], where a similar behavior of SMA with 8 wt% MA concentration was observed for PA6/PPE. It is confirmed that SMA_low_ reveals saturation content between 5 and 10 wt% where no further improvement of tensile properties is reported. SMA with high MA concentration (21.8 wt%) was shown to steadily increase the tensile properties with amounts up to 10 wt% [39]. It is noteworthy that the researchers used a PA6/PPE blend ratio of 70/30 where a droplet-sea structure was achieved independent of the SMA content. This led to a basic understanding of the effect of SMA as a compatibilizer without any further influences, such as the change in morphology. Differently, in our systems, the change in morphology suppresses the combatibilizing effect of both SMA (low and high), leading to moderate improvements (for SMA_low_) or even worsening (SMA_high_) in tensile properties.

#### Fracture Analysis

**Figure 8:**
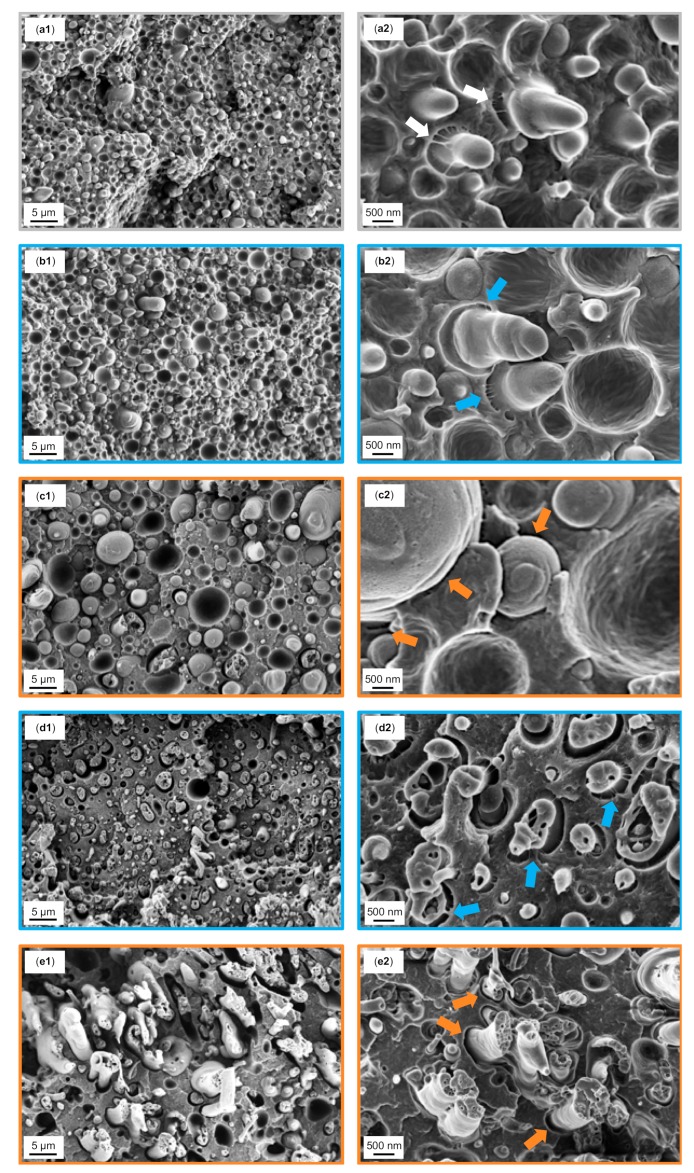
SEM fractographs after tensile tests of the reference binary blend (**a**1), 1.25 wt% SMA_low_ (**b**1) and 1.25 wt% SMA_high_ (**c**1), 10 wt% SMA_low_ (**d**1), 10 wt% SMA_high_ (**e**1), and their corresponding graphs at higher magnifications on the right column (**a**2, **b**2, **c**2, **d**2 and **e**2). The white arrows in Figure (**a**2) indicate strong matrix fibrillations, while the blue arrows in Figure (**b**2 and **d**2) indicate intermediate fibrillation, and the orange arrows in Figure (**c**2, **e**2) indicate weak interfaces.

In order to correlate the tensile properties with the fracture surface, SEM analysis of the blends after tensile testing was performed. SEM fractographs of 1.25 wt% and 10 wt% SMA_low_ and SMA_high_ are displayed together with the reference PA66/PPE blend in Figure 8a–e.

The reference (Figure 8a1,a2) shows a rough fracture surface with strong crack deflections. No plastic deformation is seen, as PA66 is rather brittle when in a dry state. The deformation behavior is predominated by pull-outs of the PPE droplets having generally insufficient interfacial bonding to the PA66 matrix. Occasionally, bound PPE domains with a low degree of plastic deformation are observed. Whenever PPE domains are elongated, matrix deformations coexist at the interface expressed by fibrillation (white arrows). Therefore, we propose that the PA66 is able to reactively couple to the hydroxyl-terminated PPE polymer. The covalent bonds allow good energy dissipation at the interfaces by debonding and fibrillation.

The fractographs of a blend with 1.25 wt% SMA_low_ (Figure 8b1,b2) show a similar surface to the prior discussed material with the brittle fracture of the PA66 matrix. In addition, pull-out of the PPE phases coexists with the fibrillar deformations of the matrix (blue arrows) where elongated PPE phases appear. Nevertheless, the tensile strength is found to be lower than the reference. Considering the high interfacial activity of SMA_low_ (see Section 3.2), we assume that an incomplete coverage of the PA66/PPE interfaces results in a disorder with lower tensile strength. More precise, the covalent bond formation between PA66 and PPE (Figure 8a2) seems to be interrupted when low amounts of SMA_low_ are added.

For 1.25 wt% SMA_high_ (Figure 8c1,c2), a smooth fracture surface is seen for having a low plastic deformation of the PA66 matrix. The PPE phase is mainly pulled-out, revealing cavities with very smooth surfaces, indicating weak interfaces (orange arrows). As SMA_high_ has a low affinity to PPE, it remains in the PA66 phase and does not disturb the formation of covalent bonds between PA66 and PPE. As for the reference, elongated PPE domains are seen, indicating locally strong interfacial bonds.

With increasing contents of SMA, a change in the morphologies is expected to result in different fracture mechanisms and thus surfaces. In Figure 8, the fractographs of 10 wt% SMA_low_ (d1, d2) and SMA_high_ (e1, e2) and are depicted. For SMA_low_ (Figure 8d1), the fracture surface with an intermediate roughness is observed. In contrast to SMA_high_ (Figure 8e1), step-like PA66 deformations (crack deflection) together with low levels of PPE elongation are very pronounced. Finding a significantly high amount of matrix fibrillation at the interfaces (Figure 8d2, blue arrows), it can be concluded that the stress transfer between both phases is very efficient. With this, the stress is deflected strongly with formation of sharp-edged steps parallel to the direction of force applied.

According to Figure 8e1 again, PA66 reveals a brittle behavior with a smooth fracture surface having weak crack deflections, whereas PPE shows a ductile behavior with cohesive failure. Typical pull-out effects are not apparent for co-continuous structures, as the phases are interpenetrating each other, acting as mechanical anchors. The PA66 exhibits further embrittlement due to dispersed SMA_high_-g-PA66 and SMA_high_ micelles. Interestingly, no interfacial bonding is observed with higher SMA_high_ content (Figure 8e2), meaning that the mechanical strength is only upheld by mechanical anchoring of the ductile PPE phase.

**Figure 9:**
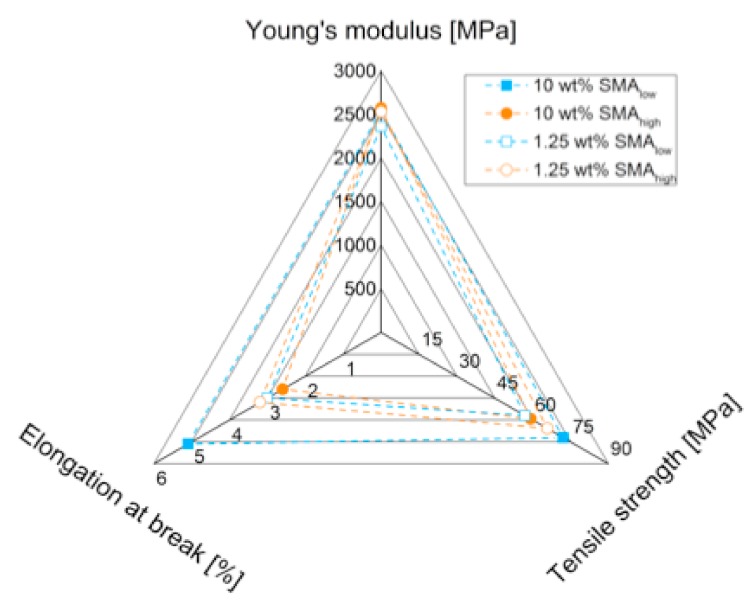
Summary of tensile properties for SMA (high and low) compatibilized PA66/PPE blend systems.

Cohesive failure of the individual blend phases is the predominant fracture mechanism for SMA_low_, whereas SMA_high_ does not show sufficient stress transfer due to weak interfaces resulting in lower tensile performance, as summarized in Figure 9.

## 4. Conclusions

In this study, we reported that PA66/PPE blends can effectively be compatibilized by using SMA copolymers. A correlation between MA concentration and SMA content and the resulting morphology and tensile properties was successfully established. Herein, it was found that SMA_high_ with higher than 8 wt% MA concentration is not miscible with PPE; thus, no interfacial interaction is observed. In contrast, SMA_low_ (8 wt% MA) revealed a complete miscibility in PPE and high interfacial activity in PA66/PPE blends. The location of the SMA, tuned by its MA concentrations, controls the morphology of the blend systems. For the immiscible SMA_high_, micellar nanostructures within the PA66 phases in the diameter range of 24 ± 8 nm were observed. These lead to an increase of the PA66 phase viscosity, thus shifting the viscosity ratio of PA66 and PPE close to 1 (for 10 wt% SMA_high_). With this, a transition from droplet-sea to co-continuous morphology for low contents of SMA_high_ (between 1.25 and 7.5 wt%) was observed. For lower MA concentrations (SMA_low_), the morphology transition is shifted to higher SMA contents starting from 7.5 wt% and ongoing for 10 wt%. With increasing SMA_low_ content, a larger number of swollen micelles were seen in the TEM micrographs. These nano-emulsions were identified to be PPE pinch-offs covered by SMA_low_-graft-copolymers with PA66. For both SMA, a droplet-sea morphology is preferred because a co-continuous morphology leads to either a plateau or a decrease in the tensile strength of the blends. In terms of tensile properties, SMA_low_ revealed the highest tensile strength of 72.5 MPa (7.5 wt%) and elongation at break of 5% (10 wt%). Enhanced tensile properties are explained by strong interfacial interaction and thus bonding between PA66 and PPE, which is expressed by the cohesive failure of the PPE phases together with strong matrix fibrillation at the interfaces. For SMA_high_, high contents were necessary to compensate for the tensile property loss. The partial recovery of the results is explained by mechanical anchoring of the PPE and PA66 phases, as a co-continuous morphology was observed for 10 wt% SMA_high_.

## Figures and Tables

**Table 1:** Properties of blend components. PA66: polyamide 66, PPE: poly (2,6-dimethyl-1,4-phenylene ether), SMA: styrene–maleic anhydride copolymers.

Material	M_w_ [g/mol]	Polydispersity [a.u.]	MA Concentration [wt%]	Supplier
PA66	60,000	1.74	-	BASF SE (Ludwigshafen, Germany)
PPE	36,800	1.98	-	Asahi Kasei K.K. (Chiyoda, Japan)
SMA_low_	245,000 ^a^	-	8 ^a^	Polyscope B.V. (Geleen, Netherlands)
SMA_high_	120,000 ^a^	-	25 ^a^	INEOS Styrolution GmbH (Frankfurt, Germany)

^a^ Values taken from technical data sheets [46].

**Table 2:** Glass transition temperatures of PPE (tan δ maximum) for reference and 10 wt% SMA contents.

Sample	T_g_ PPE	T_g_ PA66	T_g_ SMA
PA66/PPE	219	77.4	-
PA66/PPE/SMA_low_	215	71.4	130
PA66/PPE/SMA_high_	219	69.1	150

**Table 3:** Domain size distribution analysis of SMA compatibilized PA66/PPE ternary blends based on the TEM micrographs from Figure 6.

PA66/PPE	Size Distribution of Matrix Inclusions
Reference	1027 ± 336
10 wt% SMA_low_	109 ± 31 ^a^
10 wt% SMA_high_	24 ± 8 ^a^

^a^ Domain size distribution of (un-)swollen SMA-g-PA66 micelles in the PA66 matrix.
